# Combination therapy of Ulinastatin with Thrombomodulin alleviates endotoxin (LPS) - induced liver and kidney injury via inhibiting apoptosis, oxidative stress and HMGB1/TLR4/NF-κB pathway

**DOI:** 10.1080/21655979.2021.2024686

**Published:** 2022-02-11

**Authors:** Xiong Zhang, Chenlin Su, Shuxin Zhao, Ji Li, Feng Yu

**Affiliations:** Department of Basic Medicine and Clinical Pharmacy, China Pharmaceutical University, Jiangning, PR China

**Keywords:** Ulinastatin, Thrombomodulin, lipopolysaccharide, organ injury, apoptosis, anti-inflammatory, oxidative stress

## Abstract

Sepsis is a type of systemic inflammation response syndrome that leads to organ function disorders. Currently, there is no specific medicine for sepsis in clinical practice. Lipopolysaccharide (LPS) is an important endotoxin that causes sepsis. Here, we report an effective two-drug combination therapy to treat LPS-induced liver and kidney injury in endotoxic rats. Ulinastatin (UTI) and Thrombomodulin (TM) are biological macromolecules extracted from urine. In our study, combination therapy significantly improved LPS-induced liver and kidney pathological structure and functional injury, and significantly improved the survival rate of endotoxic rats. Results of TUNEL staining and Western blot showed that UTI combined with TM inhibited the excessive apoptosis of liver and kidney cells caused by LPS. The drug combination also promoted the proliferation of liver and kidney cells, reduced the levels of pro-inflammatory factors interleukin (IL)-6, IL-1β, tumor or necrosis factor (TNF)-α and nitric oxide, and down-regulated the expression of High Mobility Group Box 1 (HMGB1), Toll-like receptor (TLR) 4 and Nuclear Factor (NF)-κB phosphorylation to inhibit inflammation. In addition, the combination of UTI and TM also promoted the production of a variety of antioxidant enzymes in the tissues and inhibited the production of lipid peroxidation malondialdehyde (MDA) to enhance antioxidant defenses. Our experiments also proved that UTI combined with TM did not reduce the anticoagulant effect of TM. These results suggested that UTI combined with TM can improve endotoxin-induced liver and kidney damage and mortality by inhibiting liver and kidney cell apoptosis, promoting proliferation, and inhibiting inflammation and oxidative injury.

## Graphical abstract

**Figure:**
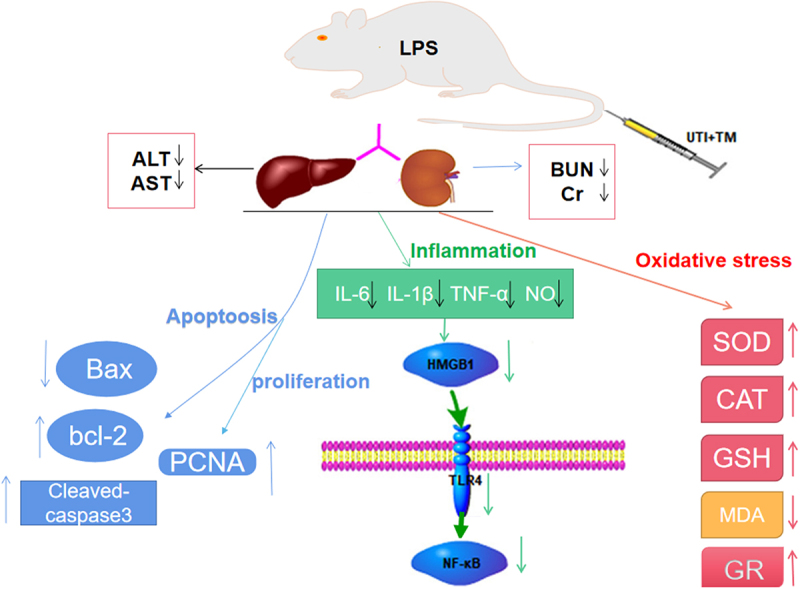

## 1. Introduction

Sepsis is a disease with a systemic inflammatory state with a known or suspected infection [1], accompanied by a high mortality rate. Endotoxic shock is the most serious form of sepsis. Society of Critical Care Medicine and European society of intensive medicine newly defined sepsis as the unbalanced response of the body to infection that leads to life-threatening organ function disorders [2,3]. The old definition of sepsis is a systemic inflammatory response syndrome caused by infection, which emphasizes infection, while the new definition of sepsis focuses on the body’s response to infection and imbalance with organ dysfunction. This definition suggests that more attention should be paid to the complex pathophysiological response caused by infection during treatment. The liver and kidney are organs vulnerable to damage from sepsis [4,5]. Acute liver and kidney injury may occur at any stage of sepsis. Sepstic patients have severe liver, and kidney injuries are often extremely critical.

An important pathogenesis of sepsis is the inflammatory response disorder of bacterial endotoxins, such as lipopolysaccharide (LPS). LPS stimulates innate immune cells to release the pro-inflammatory factor High Mobility Group Box 1 (HMGB1) [6]. HMGB1 further promotes the release of early inflammatory factors, such as tumor necrosis factor (TNF)-α and interleukin (IL)-6. When HMGB1 binds to its receptor Toll-like receptor (TLR) 4, it triggers and activates the downstream signaling molecule Nuclear Factor (NF)-κB through the transmembrane signaling pathway [7] to play an inflammatory regulatory role. In the development of sepsis, oxidative stress is like another killer that accompanies inflammation, and both are involved in the process of cell damage. Oxidative stress activates a series of transcription factors, which in turn induces the expression of a variety of genes, including a variety of pro-inflammatory cytokines. Physiological apoptosis contributes to the maintenance of the structural and functional stability of tissues and organs. However, pathological harmful factors lead to abnormal apoptosis and excessive apoptosis of cells, which is also related to organ damage caused by sepsis. Based on these situations, it is reasonable to propose that simultaneous regulation of inflammation, oxidative stress, and apoptosis, rather than a specific therapy for one of these systems, will be more effective in controlling organ injury.

Ulinastatin (UTI) is an important endogenous broad-spectrum protease inhibitor extracted from human urine. Protease inhibitors can counter-regulate the expression of proteases during inflammation to inhibit the progression of inflammation [8], UTI is considered to have essential anti-inflammatory effect. UTI is used clinically for the treatment of acute circulatory failure, acute pancreatitis and other diseases. More importantly, UTI has been determined to have a protective effect on many organs. UTI inhibits liver injury in septic mice by inhibiting inflammation and oxidation [9], UTI protects acute lung injury in septic rats by inhibiting JAK-STAT3 pathway [10].

Thrombomodulin (TM) is derived from fresh human urine too, and its recombinant product recombinant TM (rhTM) has been approved for diffuse intravascular coagulation (DIC) treatment, but a small number of studies have shown that TM also has a protective effect on sepsis-mediated organ injury, rhTM and its D1 reduce the level of histone H3, thereby alleviating the acute kidney injury [11]. In the field of liver injury caused by sepsis, the administration of recombinant TM can also improve liver dysfunction and elevate the survival rate of septic mice [12]. In a retrospective study, recombinant human soluble TM can decrease the mortality of patients with sepsis [13]. These all indicate that TM can be used in the treatment of sepsis and has a broad research prospect in organ injury.

At present, there are no specific drugs for the treatment of sepsis, and the method of combined treatment of fluid resuscitation and antibiotics is mostly used in clinic, while in animal research, the efficacy of a single drug is mostly studied [14–16]. There are few reports on the research of combined treatment. Existing research has shown that for the treatment of sepsis, drug combination therapy may have a better effect than single drug therapy [17]. The latest research also emphasized that combination therapy should be used instead of targeting single pathways or single organs [18], so it is necessary to develop a new combination therapy. Based on the fact that both UTI and TM have been found to have potential therapeutic effects on sepsis, the combination of UTI and TM may be a good drug combination choice. This combination therapy has not been reported on LPS-mediated liver and kidney injury. Whether this protective effect will be strengthened after the combination therapy, and the potential mechanism of action is worthy of in-depth exploration.

This study aims to investigate the effect of UTI combined with TM on endotoxin-induced liver and kidney injury, and whether the combination therapy exerts its function on liver and kidney injury via inhibiting cell apoptosis, promoting cell proliferation, reducing certain inflammatory mediators, inhibiting HMGB1/TLR4/NF-κB Signal transduction, inhibiting the occurrence of oxidative stress.

## 2. Materials and methods

### 2.1. Animals and treatments

Male wistar rats at the age of 7–9 weeks weighing 200–220 g were obtained from the Experimental Animal Center of Yangzhou University. The rats were held in specific pathogen-free conditions at the Animal Experiment Center of China Pharmaceutical University for at least 1 week prior to commencing studies. They were housed in a 12 h light–dark cycle-controlled room, and set at room temperature at 23–25°C and humidity (40–70%), and fed with standard laboratory diet and water. All healthy rats were randomly divided into five groups (n = 12). These groups were established as follows: Rats from Sham group received normal saline injection; rats from LPS group received 10 mg/kg LPS (Escherichia coli, 055: B5, L-2880, Source #0000110640, Batch #0000114326, Sigma, MO, USA) injection [19]; rats from the UTI group received LPS and UTI (50,000 U/kg, No. 200325, Adeal, Yangzhou, Jiangsu, China) injections; rats from TM group received LPS and TM (2000 U/kg, AD010, No. 21042701, Adeal, Yangzhou, Jiangsu, China) injections; rats from UTI + TM (UTI 50000 U/kg + TM 2000 U/kg) group received LPS, UTI and TM injections. Throughout the experiment, 1 h after LPS injection, UTI, TM or UTI+TM were injected. LPS, UTI and TM were injected into the rat body via tail vein. LPS, UTI and TM were dissolved with sterile saline before the experiment. In addition, 60 rats were grouped as described above for later survival analysis (n = 12). The protocol was approved by the Animal Research Committee of China Pharmaceutical University (Approval No.: 2021–10-005).

### 2.2. Blood, liver and kidney tissue preparation

At 6, 12, 24 hours after the injection of drugs, 5 mL of blood was collected from the orbital vein of 60 rats and centrifuged at 3000 g for 15 minutes [20]. The supernatants were collected and stored at −80°C for further use. Rats were euthanized 24 hours after drugs injection to harvest liver and kidney for subsequent experiments.

### 2.3. Biochemical analysis

The characteristic functional indicators of liver and kidney in each group were tested, and serum was taken at a 24 h time point. Blood urea nitrogen (BUN) Kit (CO13-2-1 Jiancheng, Nanjing, China), creatinine (Cr) Determination Kit (C011-2-1, Jiancheng, Nanjing, China), Aspartate aminotransferase (AST) Assay Kit (C010-2-1 Jiancheng, Nanjing, China), and Alanine aminotransferase (ALT) Assay Kit (C009-2-1, Nanjing Jiangcheng, Nanjing, China) were used to assess the activity of serum BUN, Cr, AST and ALT of each group [21,22].

### 2.4. Hematoxylin and eosin (HE) staining and histopathologic analysis

Slices of the liver and kidney were fixed for 48 h in 10% neutral buffered formalin, then dehydrated in graded concentrations of ethanol and embedded in paraffin. Samples were sectioned at 5 μm thick for HE staining [23]. The liver and kidney injuries were observed under an optical microscope with x 200 magnification (Nikon, Japan) and then photographed. Pathologists perform independent quantification scores for each specimen in a blind style. Semi-quantitative analysis of hepatocyte inflammatory cell infiltration, edema, and the necrosis in liver tissue and renal tubular dilation, mesangial cells hyperplasia in the glomerulus or renal tubular inflammation [24]. Liver injury scores. 0 points: no damage; 1 point: mild, a small amount of inflammatory cell infiltration, and a small amount of punctate necrosis; 2 points: Moderate, hepatocytes are arranged disorderly, there are some focal traits necrosis and inflammatory cell infiltration; 3 points: Severe, hepatocytes are arranged disorderly, the cell boundary is not clear, and a large number of focal necrosis and bridging necrosis and inflammatory cell infiltration are seen. Renal injury scores. 0 points: no pathological changes in the renal tubules; 1 point: mild, diseased renal tubule range <25%; 2 points: moderate, the pathological changes of the renal tubules are between 26% and 50%; 3 points: severe, the scope of the renal tubules of the disease is >50%. Each tissue is randomly observed and scored in five visual fields that do not overlap with each other.

### 2.5. Survival

After drug injection, the survival rate of rats in the Sham group, the LPS group, the UTI group, the TM group and the UTI + TM group were recorded, and the whole statistics lasted for 7 days [25].

### 2.6. Terminal-deoxynucleotidyl transferase mediated nick end labeling (TUNEL) assay

Kidney and liver tissues embedded in paraffin were sliced at 5 μm thick for TUNEL assay. The TUNEL method (KGA7071, Keygen, Nanjing, China) was used to detect cell apoptosis in liver tissues according to the manufacturer’s instructions. Finally, 2-(4-Amidinophenyl)-6-indolecarbamidine dihydrochloride (DAPI) (KGA215, Keygen, Nanjing, China) was used for mounting [26], and positive cells in the field of view were observed under an optical microscope with x 400 magnification (Nikon, Japan).

### 2.7. Enzyme-linked immunosorbent assay (ELISA)

To monitor the degree of inflammatory response of each group, serum collected at 6 h after drug injection was used. Serum levels of IL-6, IL-1β, TNF-α and nitric oxide (NO) were measured using ELISA kits (MEILIAN, Shanghai, China) according to the manufacturer’s protocol. The reaction plates were read within 15 minutes in an ELISA plate reader (Thermo Fisher,Vantaa, Finland) at 450 nm [27]. IL-6, IL-1β, TNF-α and NO concentrations were calculated relative to the appropriate standard curve, and IL-6, IL-1β and TNF-α expressed as pg/ml, NO expressed as nmol/ml.

### 2.8. Immunohistochemical analysis of proliferation of cell nuclear antigen (PCNA)

The procedure for paraffin sectioning of the liver and kidney was as described above. Paraffin sections were washed for antigen retrieval and treated with enzymatic inactivation. Then, they were blocked with 1% BSA50, incubated with the primary antibody overnight at 4 C. Goat anti-rabbit polymer (ab92552, ABCAM) was added and incubated for 20 minutes. Diaminobenzidine (DAB) solution (DAB-1031, Xinmai, Fuzhou, China) was added for color development. Hematoxylin was continually added until complete dehydration and mounting, and finally complete dehydration and mounting [28]. The percentage of 400 x PCNA positive cells was analyzed in 5 random fields in each section.

### 2.9. Western blot

Western blot was used to detect the expression of protein. Membranes were blocked with 5% TBST diluted nonfat powdered milk at room temperature for 1 hour incubated with primary antibody (HMGB1, sc-56,698, santa cruz; TLR4S, sc-293,072 santa cruz; Phosphorylation of NF(P-NF)-κB p65, sc-166,748 santa cruz; Cleaved caspase3, #9961, Cell Signaling Technology; NF-κB p65 #8242, Cell Signaling Technology; bcl-2, WL01556, Wanlei; Bax WL01637 Wanlei; GAPDH WL01114; β-actin WL01372 Wanlei.) at 4 C for 14 h. β-actin and GAPDH were used as the standard proteins. The secondary antibody was diluted with blocking solution and incubated membranes for 90 mins at room temperature [29]. Image J was used to analyze the gray value of the protein band.

### 2.10. Determination of antioxidant enzymes levels in liver and kidney

Superoxide dismutase (SOD), Glutathione peroxidase (GSH-PX), catalase (CAT) and Glutathione Reductase (GR) are the most important antioxidant enzymes that reflect the antioxidant capacity of organs. The liver and kidney tissues were made into homogenate, and the supernatant was taken after centrifuged at 2500 × g at 4°C for 15 minutes. According to the manufacturer’s instructions, the corresponding commercial kits (Jiancheng, Nanjing, China) were used to quantify the levels of GSH-PX (A005-1-1), CAT (A007-1-1), SOD (A001-2-2) and GR (A062-1-1) in liver and kidney tissues [30,31].

### 2.11. Measurement of lipid peroxidation in liver and kidney

The lipid peroxidation in the liver and kidney homogenate of experimental group rats was determined by assaying malondialdehyde (MDA) level, and the preparation of homogenate of liver and kidney tissues was as described in 2.10. According to the manufacturer’s instructions [31], the corresponding commercial kit (A003-1-1, Jiancheng, Nanjing, China) was used to quantify the levels of MDA in liver and kidney tissues.

### 2.12. Rat plasma collection and processing

After fasting overnight, blood was taken from the arteries of rats, and plasma was prepared. All plasma samples were tested within 2 h at room temperature to ensure stability. TM or UTI dissolved in physiological saline solution were mixed with rat plasma in a volume ratio of 1:99. The mixture was conducted to explore the effects of TM or UTI + TM on rat plasma coagulation indicators. At the same time, a blank control group was set. The blank control group was prepared by mixing physiological saline with rat plasma at a volume ratio of 1:99.

### 2.13. Coagulation index detection

The thrombin time (TT, Sun Biotechnology, Shanghai, China), prothrombin time (PT, international sensitivity index (ISI): 0.9; normal plasma international normalized ratio: 0.8–1.24, Sun Biotechnology, Shanghai, China) and activated partial thromboplastin time (APTT, Sun Biotechnology, Shanghai, China) detection kits were used to test these indicators using a semi-automatic coagulation factor analyzer (Zhongqingshidi, Taizhou, China) [32]. We calculate the international normalized ratio (INR) of each group of plasma according to the measured pt value, INR = (PT test/PT normal)^ISI^. The instrument parameters were set, and stirring beads were added to each channel of the test cup and the drug-containing plasma to be tested (TT: 100 μL, PT: 50 μL, APTT: 50 μL and APTT reagent 50 μL) into the test cup, placed in the 37 C pre-warming zone for 3 minutes. The test cup was transferred to the test zone, and detection reagents were added (TT: TT solution 100 μL, PT: PT solution 100 μL, APTT: CaCl_2_ solution 50 μL). Then, the results could be recorded after measuring various coagulation indexes.

### 2.14. Statistical analysis

All experiments were operated for three times, and all data were presented as mean ± standard deviation (SD). SPSS version 23.0 (IBM SPSS Statistics) and GraphPad Prism 8.0 (GraphPad Software) were used to analyze and graph all data. One-way analysis of variance (ANOVA) followed by post hoc Tukey test was used to assess the significance of statistical differences between groups. Survival rate data was analyzed by the Kaplan–Meier curve, and the log-rank statistical test was applied to compare the curves. A P-value <0.05 was considered statistically significant.

## 3. Results

Our study explored the effects of UTI combined TM on LPS-mediated liver and kidney injury and its underlying mechanism, and found that combination therapy protected the liver and kidney injury mediated by LPS. The protective effect may be related to the inhibition of cell apoptosis, promotion of liver and kidney cell proliferation, reduction of certain inflammatory mediators, inhibition of HMGB1/TLR4/NF-κB signal transduction, and inhibition of oxidative stress. It has a better effect on drug combination than drug alone. And UTI did not affect the anticoagulant effect of TM when used in combination.

### 3.1. The effect of UTI combined with TM on the characteristic indexes of liver and kidney function in rats

**Figure 1.**
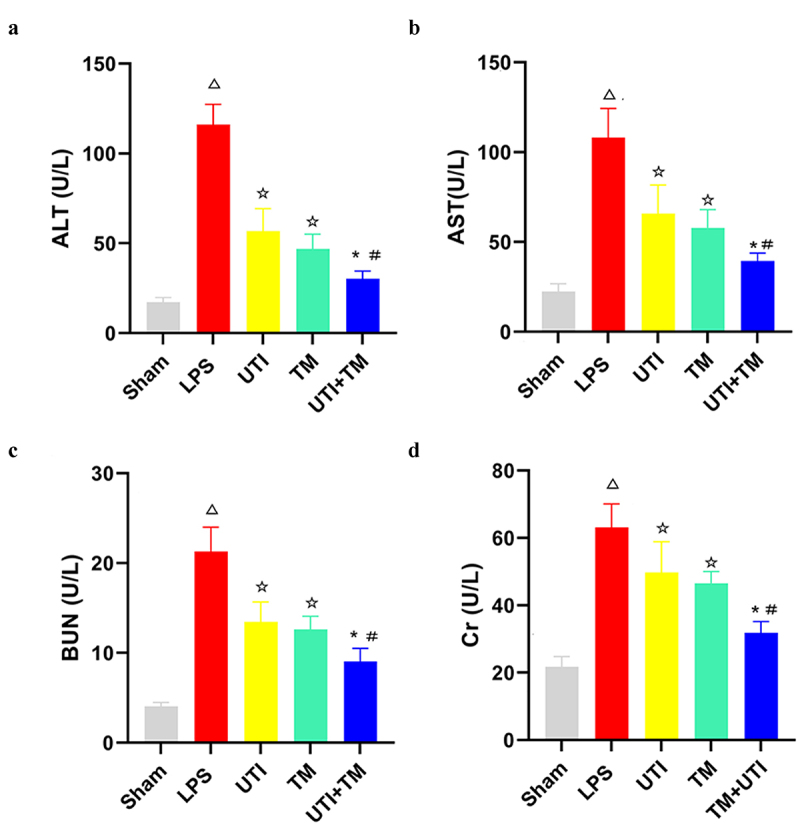
Changes of serum Alanine aminotransferase (ALT), Aspartate aminotransferase (AST), Blood urea nitrogen (BUN) and Creatinine (Cr) levels in rats. (a) serum ALT; (b) serum AST; (c)serum BUN; (d) serum Cr. *P < 0.05 vs. The UTI group. #P < 0.05 vs. The TM group, ΔP < 0.05 vs. The Sham group, ☆P < 0.05 vs. The LPS group.

ALT and AST are two vital biomarkers of liver injury. Results of the serum test showed that after the injection of LPS, compared with Sham group, ALT and AST levels of the model group increased sharply, which confirmed that the liver function of the model group was hit and damaged. 24 h after using TM and UTI, the serum ALT and AST values of rats decreased. In the UTI + TM group, ALT (Figure 1(a)) and AST (Figure 1(b)) values were further reduced, indicating that UTI combined with TM effectively restored liver function injury. The same trend was also observed in renal function indicators. Compared with the Sham group, the serum renal biomarkers BUN and Cr levels of the LPS group increased significantly, while the BUN and Cr values decreased in the TM group and the UTI group. The BUN (Figure 1(c)) and Cr (Figure 1(d)) values of drug combination group further downregulated, indicating that UTI combined with TM also effectively restored the renal function injury in endotoxic rats.

### 3.2. UTI combined with TM reduced liver and kidney pathology injury

**Figure 2.**
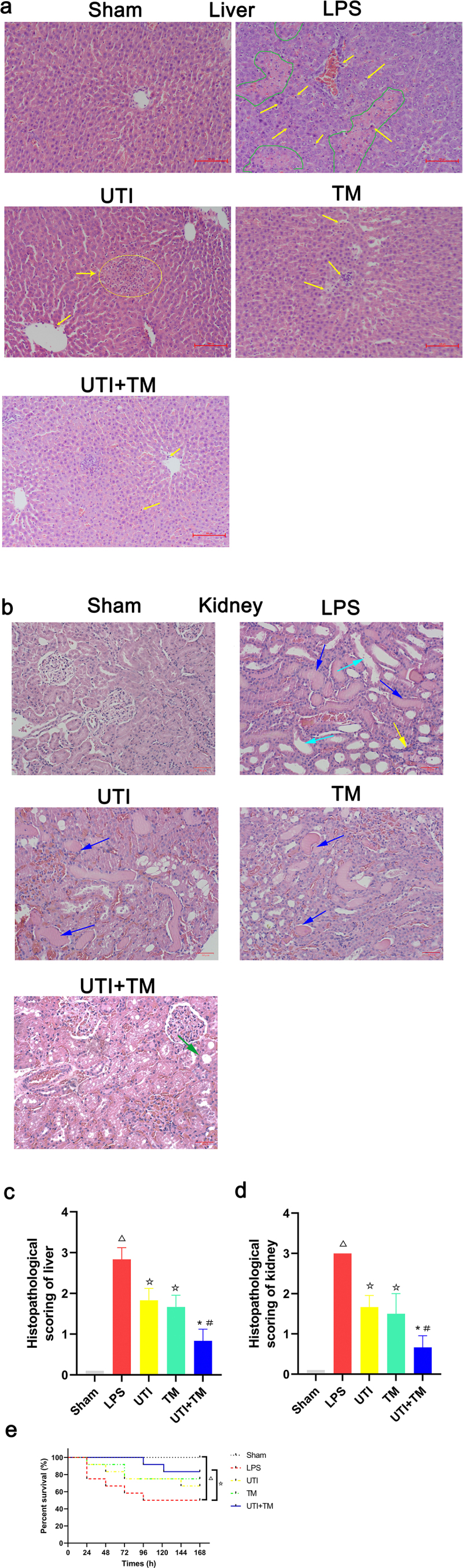
Histological changes in liver and kidney tissues of Lipopolysaccharide (LPS) induced endotoxic rats.

HE stained liver sections showed that the model group injected with LPS caused obvious liver injury, in which the liver tissue cells were irregularly arranged, accompanied by piece-meal necrosis, the disappearance of the nucleus and inflammatory cell infiltration. The liver in Sham group did not suffer any injuries. In the TM group and the UTI group, these changes were alleviated, and the cells were clear, and inflammatory cell infiltrations were observed. However, in the UTI + TM group, the liver injury was further reduced, the liver cells were clear, regularly arranged, there was no obvious edema, and the necrosis was reduced (Figure 2(a)). Kidney HE stained section, it can be observed that after injection of LPS, there are dilation of renal tubule lumen and exudation of protein mucus, mesangial cells hyperplasia in the glomerulus, and the cyst cavity disappears, a large number of inflammatory cell infiltration can be seen in the renal interstitium. The dilatation of the renal tubules in the UTI group and the TM group has been reduced, and the exudation of protein mucus still existed. The pathological damage in drug combination group improved further, and a small amount of vacuolar degenneration was observed (Figure 2(b)).

Representative pictures of HE staining of liver tissues in each group, the yellow arrows point to the inflammatory cells, the green circle point to Hepatocyte piece-meal necrosis and the disappearance of the nucleus; (b) Representative pictures of Hematoxylin and Eosin (HE) staining of kidney tissues in each group, the yellow arrows point to the inflammatory cell infiltration in the tubulointerstitium, the blue arrows point to exudation of protein mucus, the light blue arrows point to dilation of renal tubule , the green arrow points to vacuolar degeneration; (c) Score of liver tissues injury in the five groups; (D) Score of kidney tissues injury in the five groups. Score is proportional to the severity of tissues injury. (E) The effect of Ulinastatin (UTI) combination with Thrombomodulin (TM) on the number of the survival rats. n = 12 in survival rate evaluation. *P < 0.05 vs. the UTI group, #P < 0.05 vs. the TM group, ΔP < 0.05 vs. the Sham group, ☆P < 0.05 vs. the LPS group.

We also scored histological damage on HE stained sections. The higher the score displayed in the histogram, the more severe the tissue damage. A score of 0 in the Sham group means that there is no damage to the liver and kidney tissues. The liver and kidney scores of the LPS group are all greater than 2.5, which means that the tissue damage is severe, liver and kidney histology scores of the UTI group, the TM group and the drug combination group were all reduced, but the drug combination group had the lowest score (Figure 2(c)) (Figure 2(d)), which indicated that the combination of drugs has a stronger effect on the recovery of damaged tissues than UTI or TM treatment alone.

### 3.3. UTI combined with TM improved the survival rate of LPS-induced rats

The survival period of rats was monitored every 24 hours for 7 days. The rats in the Sham group showed no abnormal behavior and no death was observed. However, after injection of LPS, rats developed diarrhea, lethargy and ruffled pelage. The number of surviving rats in the LPS group decreased linearly with time, which was significantly lower than in the Sham group (P < 0.05). UTI and TM were administered separately to improve the survival rate of endotoxic rats. When UTI and TM were used in combination, the survival rate of rats had a further improvement trend (Figure 2(e)). Compared with the single-drug group, UTI + TM treatment increased the average survival rate by 33.3% (n = 12).

### 3.4. UTI combined with TM inhibited apoptosis of liver and kidney

**Figure 3.**
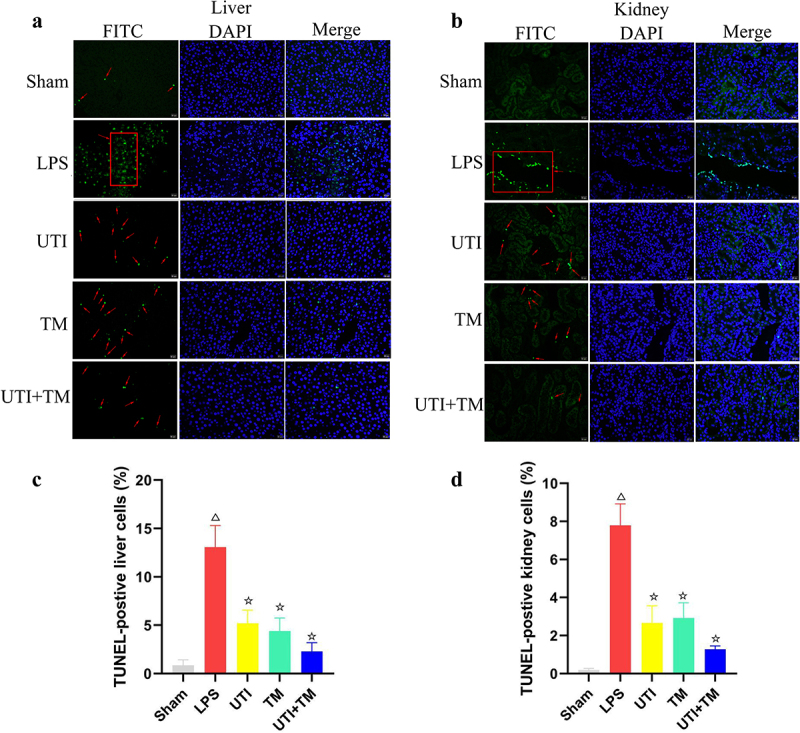
Apoptosis in liver and kidney tissues were examined by TUNEL staining.

**Figure 4.**
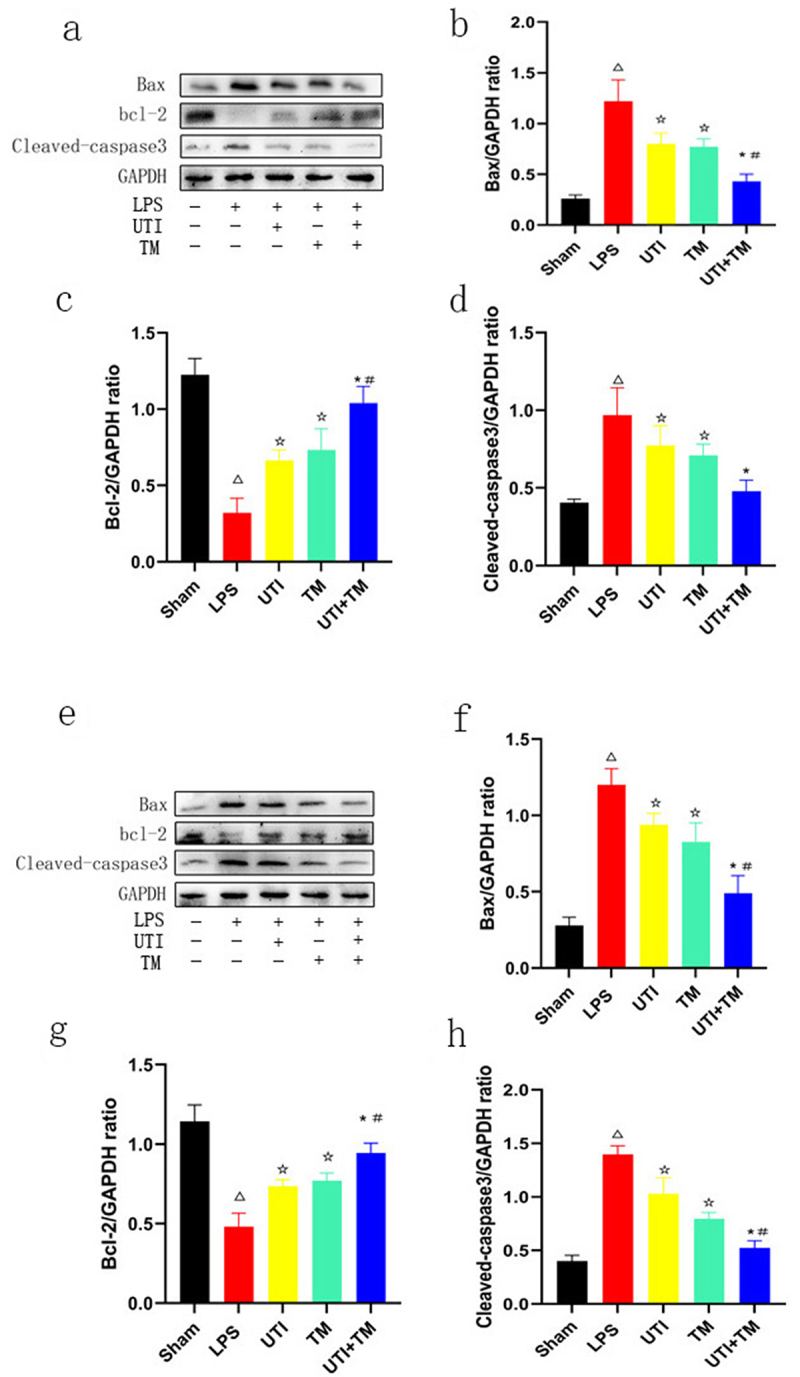
Measure the expression levels of apoptosis-related proteins in liver and kidney tissues by Western blot.

In order to clarify the protective mechanism of UTI combined with TM on liver and kidney injury in endotoxic rats, TUNEL staining was used to observe the apoptotic cells in liver (Figure 3(a)) and kidney (Figure 3(b)) slides. Compared with Sham group, positive cells in the LPS group were significantly increased, apoptosis was more likely to occur, while either UTI or TM treatment reduced the positive cells. In the UTI + TM group, the number of apoptotic cells further decreased. We also performed statistical analysis on the positive rates of liver (Figure 3(c)) and kidney (Figure 3(d)) cells in TUNEL staining. Whether in liver or kidney tissue, the apoptosis rate of the LPS group increased significantly, while after the administration of UTI and TM, the apoptosis rate appeared to decrease, and the apoptosis rate of the combination group further decreased. In addition, besides the results of TUNEL analysis, UTI and TM treatment reduced the apoptotic protein Bax and Cleaved caspase-3. When UTI and TM were used in combination, this downward trend was further accentuated; otherwise, LPS will up-regulate the expression of Bax and cleave caspase-3. The decrease in the anti-apoptotic protein bcl-2 in the LPS group was reversed by the combination of UTI and TM (liver: Figure 4(a-d); kidney: Figure 4(e-h)). These data indicated that the combination of UTI and TM can protect the liver and kidney by reducing the expression of pro-apoptotic proteins, thereby reducing cell apoptosis.

Nuclei were counterstained with DAPI (blue), and DNA fragmentation was measured by TUNEL (+) (green); the red arrows point to the apoptosis cells. (a) Representative images of TUNEL preformed on liver slices in five groups; (b) Representative images of TUNEL preformed on kidney slices in five groups; (c) Quantification of TUNEL-positive liver cells in sections of each groups; (d) Quantification of TUNEL-positive kidney cells in sections of each groups. ΔP < 0.05 vs. the Sham group, ☆P < 0.05 vs. the LPS group.

(A) Protein levels of Bax, bcl-2 and Cleaved-caspase3 in liver tissues; (B) Densitometric analyses of Bax protein in liver tissues; (C) Densitometric analyses of bcl-2 protein in liver tissues; (D) Densitometric analyses of Cleaved-caspase3 protein in liver tissues; (E) Protein levels of Bax, bcl-2 and Cleaved-caspase3 in kidney tissues; (F) Densitometric analyses of Bax protein in kidney tissues; (G) Densitometric analyses of bcl-2 protein in kidney tissues; (H) Densitometric analyses of Cleaved-caspase3 protein in kidney tissues. GAPDH was used as the control to confirm equal protein loading. *P < 0.05 vs. the UTI group. #P < 0.05 vs. the TM group, ΔP < 0.05 vs. the Sham group, ☆P < 0.05 vs. the LPS group.

### 3.5. Immunohistochemistry of PCNA

**Figure 5.**
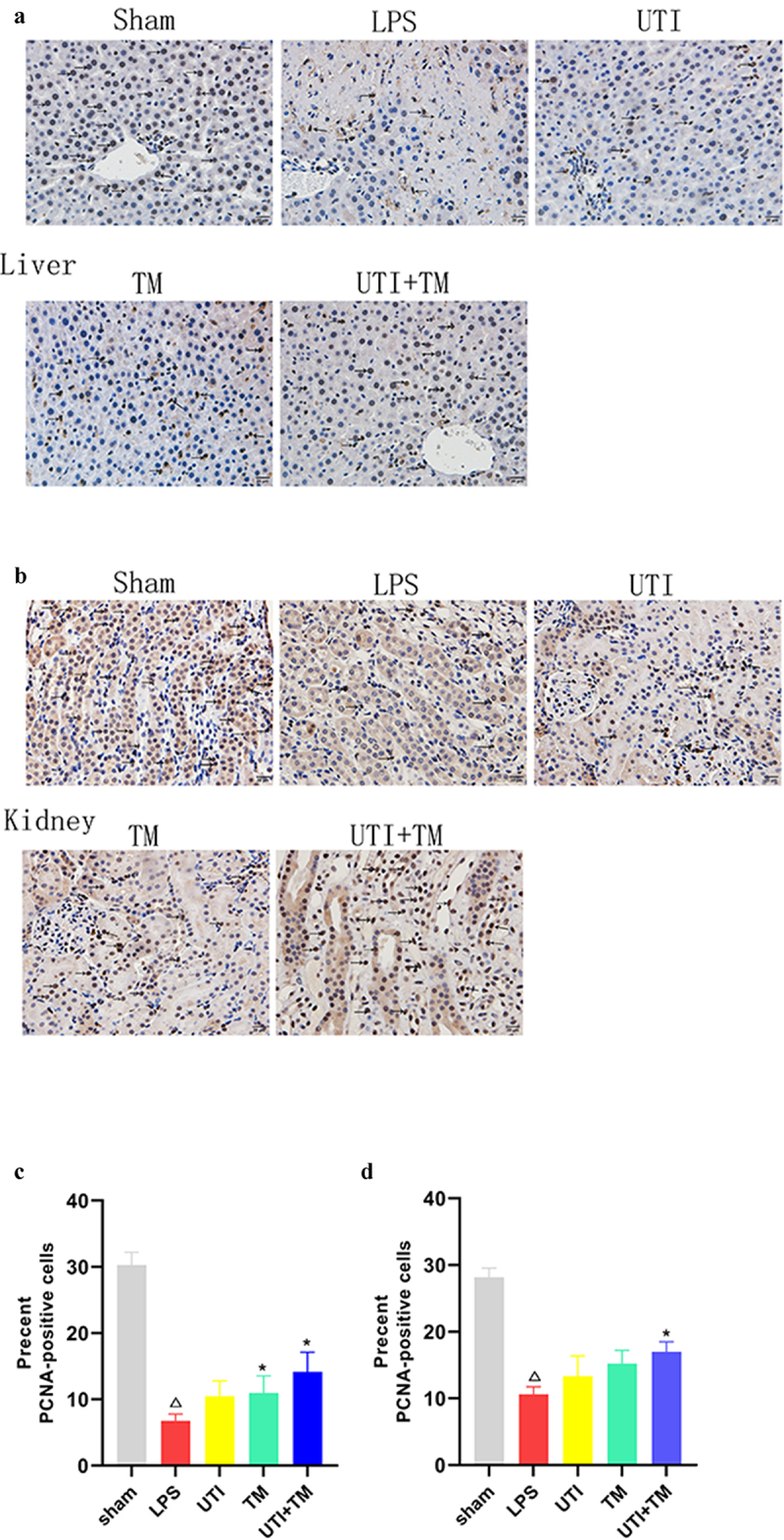
Evaluation of cell proliferation in liver and kidney tissues.

To investigate the effects of TM and UTI on the proliferation of hepatocytes and kidney cells, we performed immunohistochemical analysis on PCNA (liver: Figure 5(a)) (kidney: Figure 5(b)). We counted the number of hepatocytes in randomly taken photomicrographs. Compared with the Sham group, the ratio of positive hepatocytes/total hepatocytes decreased significantly after injection of LPS. Compared with the LPS group, in the UTI group, the positive rate increased by 3.71%, in the TM group by 4.21%, the positive rate in the UTI + TM group further increased by 7.39%. During the regeneration process, the proportion of positive hepatocytes of the UTI + TM group was greater than that of the TM group and the UTI group (Figure 5(c)). We also analyzed the PCNA of the kidney. During the whole process, the changes of PCNA in the kidney of each group of rats were consistent with the expression of PCNA in the liver. Compared with the Sham group, the ratio of positive kidney cells/total kidney cells decreased. Compared with the LPS group, in the UTI group, the ratio increased by 2.68%, in the UTI group by 4.60%, and further increased by 6.36% in the UTI + TM group (Figure 5(d)).

Immunohistochemistry was used to detect proliferation of cell nuclear antigen (PCNA)-positive cells in liver and kidney tissues. The yellow arrows point to PCNA-positive cells. (A) Representative images of PCNA of liver tissues; (B) Representative images of PCNA of kidney tissues; (C) Statistics of the positive rate of PCNA in the liver tissues; (D) Statistics of the positive rate of PCNA in the kidney tissues; *P < 0.05 vs. the UTI group, ΔP < 0.05 vs. the Sham group, ☆P < 0.05 vs. the LPS group.

### 3.6. UTI combined with TM reduced LPS-induced secretion of IL-6, IL-1β, TNF-α and NO in the serum of rats

**Figure 6.**
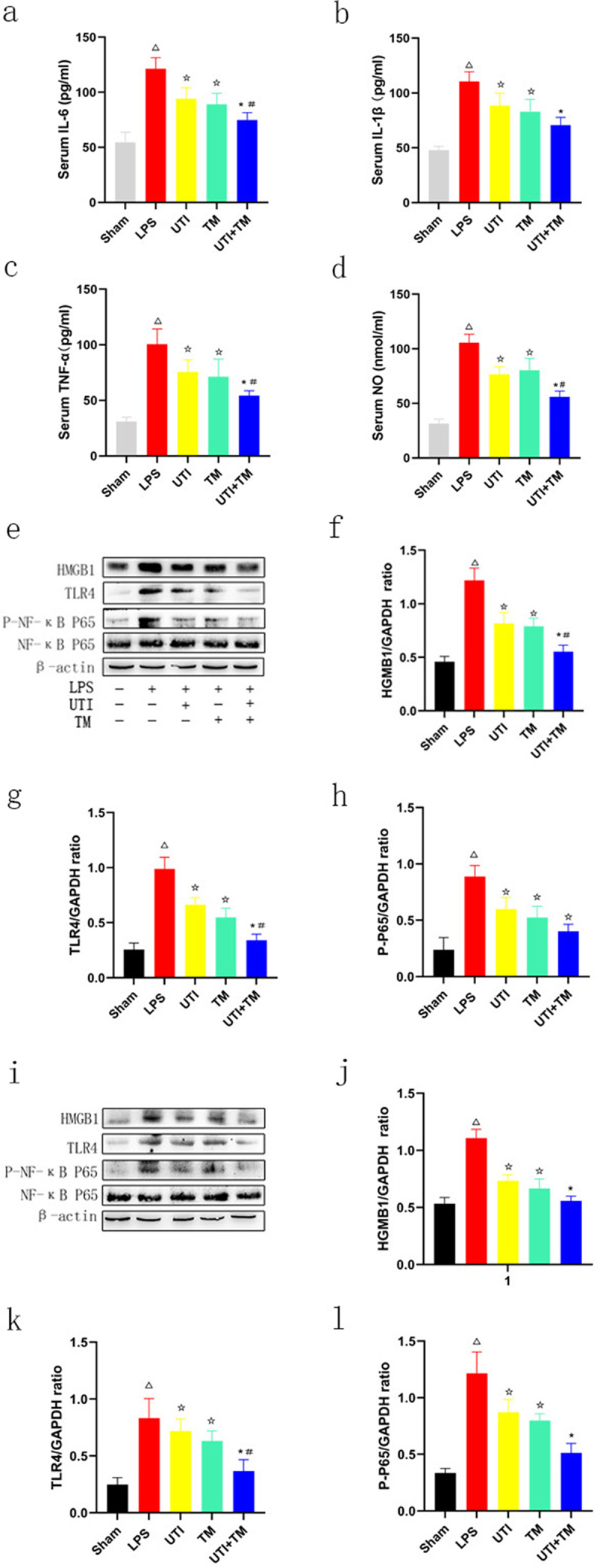
Effect of UTI combination with TM on serum cytokines and nitric oxide in rats and HMGB1/Toll-like receptor (TLR)4/Nuclear factor (NF)-κB pathway-related proteins in liver and kidney tissues.

LPS leads to the secretion of pro-inflammatory cytokines (including IL-6, IL-1β and TNF-α) in serum, and we assessed the effect of the combination of UTI and TM on the release of inflammatory factor IL-6, IL-1β, TNF-α and NO, the systematic immune status of rat after LPS injection. ELISA analysis results showed that LPS could significantly induce the increase in IL-6, IL-1β, TNF-α and NO in serum, compared with the Sham group. Both UTI and TM treatment alone reduced the serum content of IL-6, IL-1β, TNF-α and NO, and UTI combined with TM further reduced the levels of IL-6 (Figure 6(a)), IL-1β (Figure 6(b)), TNF-α (Figure 6(c)) and NO (Figure 6(d)) induced by LPS.

(a) Changes in five groups of interleukin (IL)-6; (b) Changes in five groups of interleukin (IL)-1β; (c) Changes in five groups of tumor necrosis factor (TNF)-α; (D) Changes in five groups of serum nitric oxide (NO). (e) Protein levels of HMGB1, TLR4 and NF-κB in liver tissues; (f) Densitometric analyses of HMGB1 protein in liver tissues; (g) Densitometric analyses of TLR4 protein in liver tissues; (h) Densitometric analyses of P- NF-κB protein in liver tissues (i) Protein levels of HMGB1, TLR4 and NF-κB in kidney tissues; (j) Densitometric analyses of HMGB1 protein in kidney tissues; (k) Densitometric analyses of TLR4 protein in kidney tissues; (l) Densitometric analyses of P- NF-κB protein in kidney tissues. β-actin was used as the control to confirm equal protein loading. *P < 0.05 vs. the UTI group. #P < 0.05 vs. the TM group, ΔP < 0.05 vs. the Sham group, ☆P < 0.05 vs. the LPS group.

### 3.7 UTI combined with TM inhibited TLR4-mediated NF-κB pathway

TLR4 has been identified as a receptor for LPS, and TLR4-related signal transduction pathways may mediate liver and kidney injury in endotoxic rats. In this study, in order to explore the potential mechanism of UTI combined with TM in protecting liver and kidney injury in endotoxic rats, the expression of HMGB1, TLR4 and the phosphorylation of NF-κB in liver and kidney tissues were evaluated. As shown in Figure 6 (liver:Figure 6(e-h)) (kidney: Figure 6(i-l)), compared with relative to the expression of the endogenous control β-actin, HMGB1, TLR4, and P-NF-κB proteins all increased significantly in liver and kidney tissues in the LPS group. The administration of UTI and TM remarkably inhibited the protein expression of HMGB1, TLR4 and P-NF-κB. As expected, the protein expression of HMGB1, TLR4 and P-NF-κB further decreased in endotoxic rats administered TM and UTI in combination. UTI combined with TM also significantly reduced the protein expression of downstream NF-κB.

### 3.8. UTI combined with TM inhibits oxidative stress in injured liver and kidney after LPS challenge

**Figure 7.**
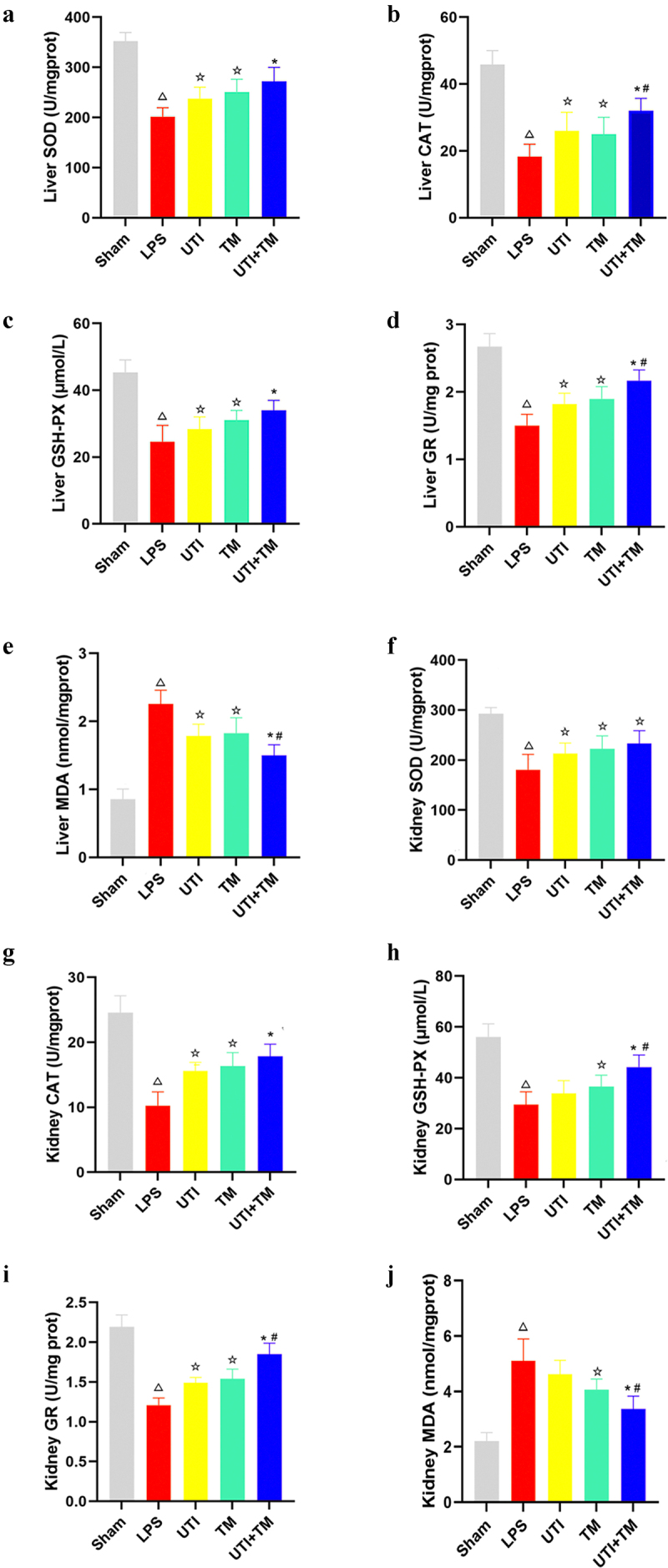
UTI combined with TM inhibits oxidative stress in injured liver and kidney after LPS challenge.

The effect on the oxidative stress parameters of the liver and kidney is shown in Figure 7. The administration of LPS significantly reduced the content of antioxidants enzymes SOD, CAT, GSH-PX and GR in liver (Figure 7(a-d)) and kidney (Figure 7(f-k)) tissues, and increased the content of lipid peroxidation MDA in liver (Figure 7(e)) and kidney (Figure 7(j)) tissues. UTI, TM, and UTI combined with TM all reversed the decrease in antioxidant enzymes SOD, CAT, GSH-PX and GR content caused by LPS, and reduced the content of MDA (liver: Figure 7(a-e); kidney: Figure 7(f-j)). More importantly, the combination therapy showed a more pronounced inhibitory effect on oxidative stress than the single drug, indicating that drug combination therapy alleviated the oxidative stress of liver and kidney in endotoxic rats.

(a) Activities of superoxide dismutase (SOD) content in liver tissues in five groups; (b) Activities of catalase (CAT) content in liver tissues in five groups; (c) Activities of Glutathione peroxidase (GSH-PX) content in liver tissues in five groups; (d) Activities of Glutathione GR content in liver tissues in five groups; (e) Activities of malondialdehyde (MDA) content in liver tissues in five groups; (f) Activities of SOD content in kidney tissues in five groups; (g) Activities of CAT content in kidney tissues in five groups; (h) Activities of GSH-PX content in kidney tissues in five groups; (i) Activities GR content in kidney tissues in five groups; (J) Activities of MDA content in kidney tissues in five groups. *P < 0.05 vs. the UTI group. #P < 0.05 vs. the TM group, ΔP < 0.05 vs. the Sham group, ☆P < 0.05 vs. the LPS group.

### 3.9. Influence of UTI on the anticoagulant function of TM

As shown in Table 1, as expected, in the range of 0–200 U/mL, TT, PT INR and APTT upregulated with the increase of TM concentration, and all had a good linear correlation (r^2^ > 0.98). TT was extremely sensitive to TM. Compared with the blank control group, 200 U/mL TM significantly prolonged TT by about 3.23 times (P < 0.05). However, the increase of UTI concentration in the range of 0–200 U/mL did not extend the TT, PT, INR and APTT of rat plasma (P < 0.05, there was no statistical difference in anticoagulant effect). Based on the above results, 100 U/mL of TM was selected for subsequent experiments with UTI (100 U/mL) to investigate whether UTI would reduce the anticoagulant effect of TM when combined. Importantly, when UTI was used in combination with TM, TT, PT, INR and APTT were not shortened compared with the TM group, which indicated that UTI does not affect the anticoagulant effect of TM when they are used in combination (Table 1).

**Table 1.** Effects of UTI and TM on coagulation indicators of rat plasma

Indicator	c/(U/mL)	TT/s	PT/s	INR	APTT/s
Control	0	41.52 ± 0.66	18.10 ± 0.29	1	37.75 ± 0.58
UTI	50	42.11 ± 0.65	18.27 ± 0.35	1.008 ± 0.01	38.65 ± 0.79
	100	41.33 ± 1.25	18.85 ± 0.46	1.037 ± 0.01	38.18 ± 0.94
	150	42.17 ± 0.66	18.83 ± 0.65	1.036 ± 0.03	39.35 ± 0.77
TM	50	81.57 ± 1.14*	22.03 ± 0.52*	1.194 ± 0.04*	51.32 ± 0.97*
	100	114.23 ± 1.97*	24.02 ± 0.59*	1.295 + 0.04*	69.33 ± 1.07*
	150	147.10 ± 2.18*	29.18 ± 0.62*	1.537 ± 0.01*	79.45 ± 1.31*
UTI+TM	100UTI+100TM	119.21 ± 3.31*^n^	24.20 ± 0.82*^n^	1.298 ± 0.03*^n^	70.78 ± 0.87*^n^

*P < 0.05 vs. 0 μg/mL group; n: no significant vs. 100TM; UTI: ulinastatin; TM: thrombomodulin; TT: thrombin time; PT: prothrombin time; INR: international normalized ratio; APTT: activated partial thrombin time

## 4. Discussion

Sepsis is a systemic inflammatory response syndrome, and endotoxin shock is one of its most serious manifestations [33]. At the same time, sepsis affects various systems and organs of the body. It causes damage to cells and tissues and affects metabolism, which further leads to the failure of various vital organs [34,35]. Therefore, protecting organs from damage and repairing organ injury is particularly important. The effects of the existing treatment strategies for organ injury are not satisfactory. In consequence, new treatments or drugs are urgently needed. UTI and TM are glycoproteins extracted from human urine. Several studies have reported that these two drugs have significant anti-inflammatory effects and a certain protective effect on liver and kidney injury. Therefore, we further tested the protective effect of UTI combined with TM on endotoxic rats.

LPS is a component in the outer wall of gram-negative bacteria [36]. Injecting LPS into animals can produce endotoxin shock pathological changes, and endotoxic shock is one of the most serious manifestations of sepsis. Therefore, LPS-induced endotoxic model is widely used to discover drugs and treatment tools for sepsis caused by Gram-negative bacterial infections [37]. It can activate mononuclear macrophages and endothelial cells through the cell signal transduction system in the body, synthesize and release a variety of inflammatory mediators [38], which in turn cause a series of reactions to the body. Pro-inflammatory cytokines such as IL-6 and TNF-α are involved in the initiation and regulation of the inflammatory response [39]. It has been confirmed that a large amount of TNF-α and IL-6 are produced in macrophages exposed to LPS [40]. In our research, serum pro-inflammatory factors TNF-α and IL-6 were significantly increased in rats injected with LPS. However, UTI combined with TM significantly inhibited the levels of TNF-α and IL-6 in serum, indicating that its protective effect on endotoxic rats may be related to its anti-inflammatory properties.

HMGB1/TLR4/NF-κB is an important inflammatory signal pathway in LPS-induced inflammation [41]. Some reports have shown that HMGB1/TLR4 common syndrome pathway genes are expressed in the liver and kidney [42,43]. HMGB1 is a highly conserved non-histone DNA binding protein, which is widely distributed among various organs, such as lung, brain, liver, heart, and kidney. HMGB1 can be released from necrotic cells through active secretion and passive release, inducing inflammation. HMGB1 is also one of the endogenous ligands of TLR4, which is also widely expressed in the liver and kidney. LPS induces tissues to release HMGB1, which mediates autophagy or triggers the initiation of inflammation through the TLR4 signaling pathway, triggering a series of cascade reactions. It mainly includes two pathways, including the myeloid differentiation factor 88 (MYD88) dependent pathway and TRIF dependent pathway [44]. Activation of MYD88 activates downstream IKK-α/IKK-β, leading to the phosphorylation and degradation of IκB-α, and finally NF-κB is activated [45]. Phosphorylation of NF-κB leads to the release of pro-inflammatory cytokines, including TNF-α, IL-1β and IL-6. Therefore, inhibiting HMGB1-TLR4 signaling pathway may effectively improve organ injury mediated by endotoxin. Our research has confirmed that UTI and TM could significantly inhibit LPS-induced liver and kidney injury through the HMGB1/TLR4/NF-κB pathway. When UTI and TM were used in combination, this effect was more significant, and it also upregulated the survival rate of rats attacked by LPS.

Oxidative stress generally occurs with inflammation. Oxidative stress activates a series of transcription factors, and then induces the expression of various genes, including a variety of pro-inflammatory cytokines. After oxidative stress causes organ damage, the body’s antioxidant defense system can inhibit the production of free radicals, further scavenging free radicals, and induce antioxidant enzymes to play a protective role. SOD, GSH-PX, and CAT are all important antioxidant enzymes, which can effectively scavenge free radicals, inhibit the formation of lipid peroxidation reactants, and protect the body [46]. MDA is the end product of free radicals involved in the lipid peroxidation reaction. Its abnormal expression can damage the membrane system, aggravate the degree of tissue damage, and cause cell degeneration or even necrosis. A large number of studies have described the oxidative stress of patients with sepsis and the existence of antioxidant depletion [47]. Our research has also verified this point. In LPS-mediated sepsis rats, the contents of antioxidant enzymes SOD, CAT and GR all have abnormally decreased, and MDA has increased, indicating that oxidative stress damage occurred during sepsis. After combined therapy treatment, it can reduce the content of MDA and increase the content of SOD, GSH-PX, GR and CAT. These findings suggest that the regulation of free radicals in lipid peroxidation reactions and antioxidase by combination therapy was involved in its beneficial action against LPS-induced liver and kidney injury.

It is worth noting that LPS promotes apoptosis of the liver and kidney cells and aggravates tissue injury [48]. The results of Western blot confirmed the protein concentration of Cleaved caspase-3 and Bax increased, and the concentration of anti-apoptotic protein bcl-2 decreased. In serum, ALT and AST, as indicators of liver characteristics, as well as BUN and Cr, as indicators of kidney characteristics, were increased. It showed that the action of endotoxins led to apoptosis of liver cells and kidney cells. With the administration of UTI and TM, the concentration of Cleaved caspase-3 and Bax decreased, the concentration of bcl-2 increased, and the values of ALT, AST, BUN and Cr began to decrease. All these indicated that liver and kidney injuries were alleviated, which were further alleviated when UTI combined with TM. Furthermore, the TUNEL results confirmed the consistent results. The number of positive cells in liver and kidney tissues decreased after UTI combined with TM, which reversed the increase in the number of positive cells caused by LPS. We also observed the changes in the number of PCNA-positive cells in the liver and kidney tissues. The changes in PCNA were usually closely related to tissue regeneration [49]. After LPS injection, the PCNA of rat liver and kidney tissues decreased significantly. LPS exposure would affect these proliferating cells and cause a decrease in their number due to cell death. After the administration, PCNA began to increase, and the combination group’s level was higher than LPS group. Interestingly, we observed that at the concentration we set, the single-drug groups increased in proliferation but not significantly compared to the LPS group. This indicates that combination therapy does promote cell proliferation more than a single administration.

The blood coagulation system plays an important role in the pathogenesis of sepsis. It is mutually reinforcing with inflammation, and together constitutes a key factor in the occurrence and development of sepsis [50]. Endotoxin activates the exogenous coagulation pathway by inducing the release of tissue factor of macrophages and endothelial cells. The coagulation factor XII activated by endotoxin can also further activate the endogenous coagulation pathway, which ultimately leads to disseminated intravascular coagulation (DIC) [51,52]. Therefore, we envision that in the treatment of DIC induced by sepsis, TM can not only effectively treat sepsis but may inhibit the symptoms of DIC by activating the anticoagulation system. Our experiments proved that compared to using TM alone, UTI combined with TM did not reduce the anticoagulant effect of TM. We concluded that the combined use of UTI and TM was effective in the treatment of sepsis, and UTI did not affect the anti-DIC effect of TM. Therefore, the combination of UTI and TM is a good strategy when selecting drugs in the special population of sepsis-mediated DIC.

At present, the exploration of combination therapy to protect against sepsis-mediated organ injury has never stopped. Previous studies have shown that the combination of sodium ferulate and oxymatrine could protect lung and liver injury in septic mice by reducing systemic inflammation and reducing oxidative damage [53]. Clindamycin combined with ceftriaxone improved the survival rate of septic mice and prevented organ injury through immunomodulatory effects [54], and melatonin combined with irisin ameliorated LPS-induced cardiac dysfunction via inhibiting the Mst1-JNK pathways [55]. Coenzyme Q10 combined with aescin inhibited NLRP-3 inflammasome by regulating mitochondrial stability, thereby preventing LPS-induced acute lung injury in septic rats [56]. In addition to the combination of different drugs, the combined use of stem cells and drugs has also been proven to have a certain effect on the treatment of sepsis. Menstrual-derived mesenchymal stem cells combined with antibiotics could improve the sepsis-mediated liver damage and increase the survival rate of sepsis by reducing the pro-inflammatory and anti-inflammatory cytokines to regulate the inflammatory response [57]. The combination of gases for treatment is also an interesting research direction. The combined therapy of molecular hydrogen and hyperoxia has been proved to have a therapeutic effect on sepsis. The combination therapy of H_2_ and hyperoxia enhanced the therapeutic effect through antioxidant and anti-inflammatory mechanisms, and has a protective effect on lung, liver and kidney injury in mice [58]. These studies have shown that the mechanism of the protective effect of combination therapy on organ injury in sepsis is diverse, mainly by inhibiting inflammation and oxidative stress, and regulating the immune system to play an organ protective effect. Our research further confirmed the importance of inhibiting inflammation and oxidative stress in the treatment of sepsis. For the first time, we proposed and verified the protective effect of promoting cell proliferation on promoting liver and kidney, and for the first time, it was confirmed that UTI and TM exert anti-inflammatory effects by inhibiting the activation of the HMGB1/TLR4/NF-κB pathway. Existing studies have also given us new ideas whether the combined effect of UTI and TM is to inhibit NLRP-3 inflammasome or inhibit the Mst1-JNK pathway by regulating mitochondrial stability, and the mechanism through regulation of the body’s immune system deserves further investigation.

Our research explored the feasibility of UTI combined with TM for the first time, and found that the combination therapy has a protective effect on the liver and kidney at the same time, and also found that this protective effect is partly achieved by promoting cell proliferation. In terms of anti-inflammatory mechanism, it has been further discovered that the combination of drugs has a regulatory effect on HMGB1/TLR4/NF-κB pathway. It was also found that the combination of UTI and TM would not affect the anticoagulant effect of TM. It is undeniable that the study still has limitations. First, our experiments did not conduct further studies into the damage and repair of other important organs, such as the heart and lungs. In addition, we have confirmed that UTI combined with TM does have better efficacy than single drugs, but there is no further research on the combination of UTI and TM to achieve the same therapeutic effect to reduce the dose of single drugs. These are all worthy of further exploration.

## 5. Conclusion

Our research showed that UTI and TM combination therapy protected the liver and kidney of LPS-treated rats. These protective effects can be attributed to UTI and TM inhibiting apoptosis, promoting the proliferation of liver and kidney cells, reducing certain inflammatory mediators such as TNF-α, IL-6, inhibiting HMGB1/TLR4/NF-κB signaling, and inhibiting the occurrence of oxidative stress. The protective effect of endotoxic liver and kidney injury and inflammation provided new insights into the treatment of liver and kidney injuries caused by endotoxin. Therefore, considering these results, the combination of TM and UTI may be a promising strategy for the treatment of endotoxin-mediated organ injury.

## Declare

We save our manuscript on the preprint server ‘research square’ (DOI:10.21203/rs.3.rs-817,705/v1) (https://www.researchsquare.com/article/rs-817705/v1). The preprint does not mean that the article has been published. It has not been published in any form of journal. Its copyright still belongs to the authors. We agree to transfer the copyright of the article to Bioengineered.
